# Origami Lesion-Targeting Device for CT-Guided Interventions

**DOI:** 10.3390/jimaging5020023

**Published:** 2019-01-23

**Authors:** Austin J. Taylor, Sheng Xu, Bradford J. Wood, Zion Tsz Ho Tse

**Affiliations:** 1 School of Electrical and Computer Engineering, University of Georgia, Athens, GA 30602, USA; 2 Center for Interventional Oncology, National Institutes of Health, Bethesda, MD 20892, USA; 3 3T Technologies, LLC., Marietta, GA 30067, USA

**Keywords:** origami, percutaneous biopsy, computed tomography, radiologic phantom, 3D printing

## Abstract

The objective of this study is to preliminarily evaluate a lesion-targeting device for CT-guided interventions. The device is created by laser cutting the structure from a sheet of medical grade paperboard, 3D printing two radiocontrast agent grids onto the surface and folding the structure into a rectangular prism with a viewing window. An abdominal imaging phantom was used to evaluate the device through CT imaging and the targeting of lesions for needle insertion. The lesion-targeting trials resulted in a mean targeting error of 2.53 mm (SD 0.59 mm, *n* = 30). The device is rigid enough to adequately support standard biopsy needles, and it attaches to the patient, reducing the risk of tissue laceration by needles held rigidly in place by an external manipulator. Additional advantages include adequate support for the insertion of multiple surgical tools at once for procedures such as composite ablation and the potential to guide off-axial needle insertion. The low-cost and disposability of the device make it well-suited for the minimally invasive image-guided therapy environment.

## 1. Introduction

Image-guided percutaneous biopsy has become an essential practice in modern medical care. It has been shown to be an effective, safe and reliable technique [1,2,3,4] to puncture lesions or provide access for drainages. Nevertheless, various complications still arise, including hemorrhaging, hematomas, and injury to surrounding anatomical structures [5,6,7,8,9,10]. The risk of complications may be amplified if the lesion location is difficult to reach or if patient positioning is suboptimal.

Traditionally, CT-guided procedures are performed using the freehand technique. This can be time-consuming, especially if the target is small or difficult to reach. In these scenarios, numerous needle insertions and CT scans may be required, thus increasing time, risk, needle manipulations, tissue damage, and radiation dosage. The development of guidance, navigation and robotic systems has dramatically improved the safety and efficacy of image-guided interventional procedures. Various laser guidance systems [11,12,13] have aimed to improve the puncture accuracy, but these systems require the needle to stay axially aligned with the laser beam throughout the insertion and do not provide physical support to hold the needle in place. Other guidance systems have aimed to address these issues but are only able to accommodate a single needle [14,15,16]. Most currently used navigation systems use electromagnetic [17,18,19,20,21,22,23,24,25,26,27] or optical tracking systems [28,29,30,31]. Robotic positioning and guidance platforms such as the AcuBot, B-Rob, INNOMOTION, the Mitsubishi RV-E2 lung biopsy robot, the KUKA LWR robot, the ROBIO EX, and the iSYS1 robot system provide accurate and stable needle guidance [31,32,33,34,35,36,37,38,39,40] and are especially useful in the case of limited space at the skin entry site or a difficult angulated access. The main drawbacks in computer-navigated and robotic interventions include high costs for the development of such systems, complex operation of the devices, and increased operation time due to additional procedures including system set-up, instrument calibration, registration, and verification of accuracy. Thus, familiarity with these systems is important for routine and fast use. For the application of most computer navigated and robotic systems, an additional person (technician) is helpful, and the costs for the purchase of the system (approximately 100–300,000 $) and the additional personnel are often unaffordable for smaller hospitals.

In this study, we present a disposable origami lesion-targeting device that provides a cost-effective method for holding and guiding a biopsy needle to a target location during CT-guided interventions. The device removes the need for additional navigation software and patient registration. The proposed solution was evaluated in an abdominal phantom, and we present results of the device workspace and the lesion-targeting accuracy.

## 2. Materials and Methods

### 2.1. Origami Lesion Targeting Device Design

The design of the origami lesion-targeting device originated from specifications suggested by surgeons and MRI interventional radiologists. Suggestions included:

- Disposable or able to be sterilized for future use
- Attached securely to patient
- Allow for in-plane or out-of-plane needle insertions
- Support the insertion of multiple needles
- The device should not require additional software

**Figure 1:**
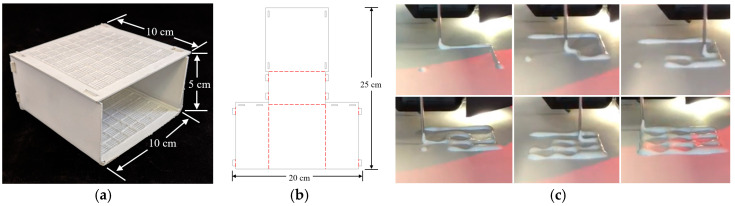
(**a**) Origami lesion targeting device. (**b**) Device folding pattern; solid black lines represent cut lines and dashed red lines represent fold lines. (**c**) 3D printing RCA mixture.

A template-based guidance system was determined to be the most effective way to satisfy the suggested design specifications. The origami lesion-targeting device (Figure 1a) is constructed by laser cutting an origami folding pattern (Figure 1b) from solid bleached sulphate, a virgin fiber grade of paperboard. The thickness of the paperboard can be adjusted to ensure no bending of the plane occurs upon needle insertion. An RCA grid (Figure 1c) is 3D printed onto the surface of the paperboard and given time to dry before the board is turned over and another RCA grid is printed. Once both RCA grids have dried, the device is then folded into its final form. Double sided mounting tape was applied to the bottom of the device to provide an effective method for mounting the device securely to the patient.

The folding pattern is designed with computer-aided design (CAD) software, AutoCAD, and cut with a Full Spectrum laser cutter. The folding pattern design consists of five panels which fold to form a 50 mm × 100 mm × 100 mm rectangular prism with one side left open to serve as a viewing window. The dimensions can be modified to accommodate the intended application. The folded panels are held in place with eight tabs which fit snuggly into the designated slots.

### 2.2. Radiocontrast Agent Mixture

The RCA mixture was developed with three major design criteria in mind. The first and most important goal was for the mixture to be bright enough to show up in a CT scan. Secondly, the mixture needed to be applied to a surface as a liquid and be able to dry into a solid. Lastly, we designed the RCA mixture to have a viscosity which would allow it to flow through a 1 mm orifice for extrusion via a syringe in a controlled manner. The resulting mixture was 70% Elmer’s glue, 20% water, and 10% barium sulfate measured by mass. Barium sulfate, Hi-LR from HiMedia Laboratiories was chosen as the radiocontrast agent due to its exceptional radiopacity [41].

### 2.3. 3D Printing Radiocontrast Agent

To increase manufacturability and to decrease fabrication time, the RCA mixture grid was 3D printed onto the surface of the device. The lines of the RCA grid were printed in 1-cm increments, and additional 2-mm gridlines were raster engraved into the surfaces to provide an effective means of measuring needle insertion locations. A Fisher Scientific syringe pump was used to extrude the mixture from an EXELINT 50-ml Luer Lock Tip syringe at a constant rate. A 5-mm diameter tube connected the syringe to the side of an extruder head of a MakerBot 3D printer (MakerBot, New York City, NY, USA). The mouth of the tube was aligned vertically with the 3D printer nozzle so that both contacted the printing surface at the same time. A 3D printing job was run on the printer which moved the extruder head along the path of the desired grid. The syringe pump was manually turned on and off when the print job started and finished.

### 2.4. Lesion Targeting Equations

**Figure 2:**
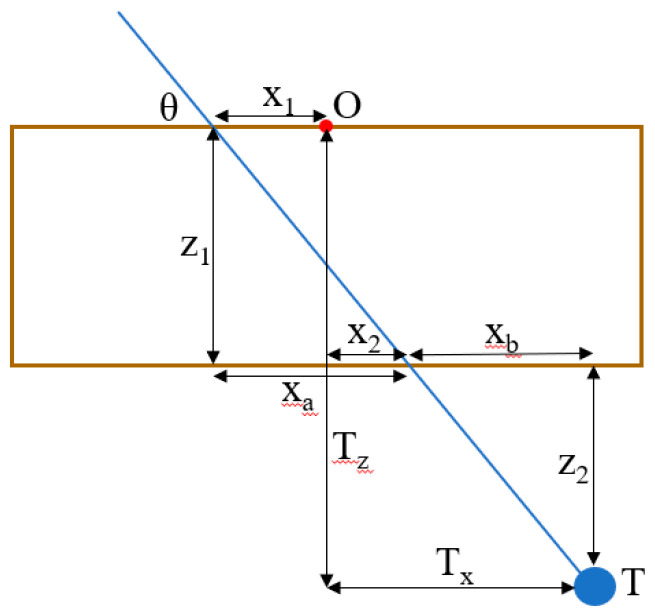
Schematic diagram of a needle insertion trajectory.

Figure 2 depicts a 2D schematic diagram of a needle trajectory through the device to a target lesion. From the schematic, several equations can be derived to determine the proper coordinates (*x*_1_, *y*_1_, *x*_2_, *y*_2_) in the top and bottom layers of the device through which to insert the needle to hit the target lesion. Table 1 defines the variables used in the targeting equations. From a CT scan, the physician can determine the desired insertion angle *θ* and the approximate width, length, and depth (*T_x_*, *T_y_*, *T_z_*) the target is from the origin of the device. Given the target depth *T_z_*, the vertical distance from the target layer to the bottom layer *z_2_* can be found by

(1) $$z_{2}=T_{z}-z_{1}$$

Knowing the insertion angle, the horizontal distance from the second insertion location to the target *x_b_* can be found by

(2) $$x_{b}=\frac{z_{2}}{\tan\theta }$$

The target width *T_x_* is the sum of the horizontal distance from the origin to the insertion location on the bottom layer *x*_2_ and the horizontal distance from the bottom layer insertion location to the target *x_b_*, so *x*_2_ can be found by

(3) $$x_{2}=T_{x}-x_{b}$$

The horizontal distance from the origin to the top layer insertion location can be found in a similar manner by

(4) $$x_{a}=\frac{z_{1}}{\tan\theta }$$

(5) $$x_{1}=x_{a}-x_{2}$$

The insertion coordinates *y*_1_ and *y*_2_ can be found using the same equations, replacing *x* with *y* and *T_x_* with *T_y_*. The insertion depth can be found by

(6) $$d=\sqrt{{\left(x_{a}+x_{b}\right)}^{2}+{\left(z_{1}+z_{2}\right)}^{2}}$$

### 2.5. Workflow

The workflow of a typical procedure using the device is described below.

1. Perform a diagnostic CT or ultrasound scan of the target area to locate the target lesion to determine positioning of the patient and the approximate skin entry point or region.
2. Place the needle guide on the patient and perform another CT scan to visualize the location of the target lesion with respect to the needle guide.
3. Measure the approximate transverse, axial, and sagittal distances from the origin of the needle guide to the target lesion on the CT console or workstation.
4. Use the needle insertion location Equations (1)–(6) to determine the insertion locations and the insertion depth. (This step may be semi-automated)
5. Insert the needle into the calculated locations of the needle guide by measuring the distance from the origin using the 1 cm spacing between the gridlines, stopping insertion just after traversing the skin.
6. Perform another CT scan in the same respiratory cycle to confirm the needle is aligned with the target lesion. If yes, continue to step 7. If no, repeat steps 3–6.
7. Continue pushing the needle for the entire calculated insertion depth to contact the target lesion.
8. Perform another CT scan to confirm the target lesion is on track to be sampled (depending upon forward throw gun versus one snap gun). If yes, collect the sample and remove the needle from the patient. If no, retract the needle and repeat steps 6 & 7.

### 2.6. Validation of Targeting Accuracy

**Figure 3:**
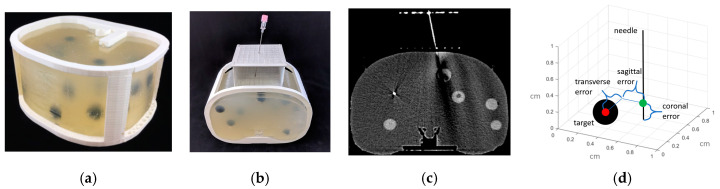
(**a**) 3D printed abdominal phantom for quantification of the lesion-targeting error. (**b**) Needle being inserted into the phantom using the device. (**c**) CT image showing needle insertion. (**d**) Targeting errors were measured in transverse, sagittal and coronal directions of the needle relative to the target lesion.

An abdominal phantom was used to perform lesion-targeting experiments (*n* = 30) to validate the accuracy of the needle guide (Figure 3a). The phantom consisted of a 3D printed outer shell with a soft plastic filling designed to match the density of human fatty tissue. The phantom contained various soft 3D printed tumors located throughout the abdominal cavity which were used as targets for needle insertions. The needle guide was positioned in the ventral insertion window of the phantom and the origin of the needle guide was aligned with the CT laser to assure proper craniocaudal angulation. After initial scans, the insertion locations and the insertion depth were calculated, and the biopsy needle (18-gauge × 200 mm) was inserted. Using confirmation scans, transverse, sagittal and coronal distance errors were calculated from the coordinates of CT images based on the distance between the needle tip and the center of the target tumor (Figure 3d). Total error is calculated as the root mean square (RMS) distance of the transverse, sagittal and coronal errors.

## 3. Results

### 3.1. Workspace Analysis

**Figure 4:**
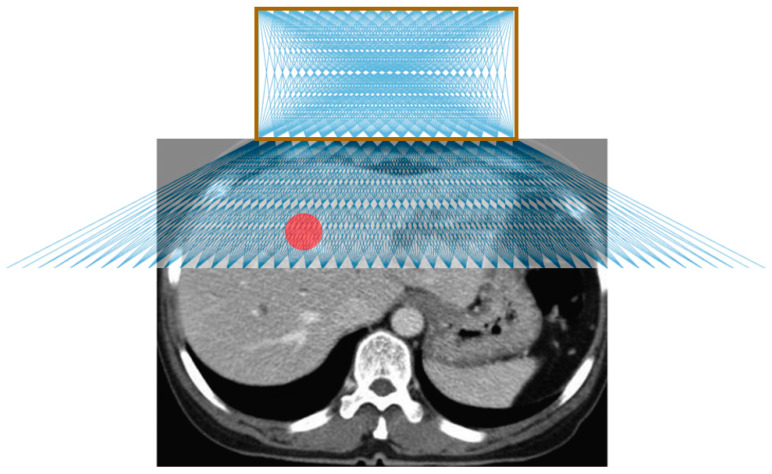
Illustration of possible needle trajectories in the transverse plane. The brown square represents the lesion-targeting device, the red circle represents the target lesion, and the blue lines represent the needle trajectories.

Figure 4 shows the possible needle trajectories in the transverse plane provided by the gridlines on the origami lesion-targeting device. The discrete potential insertion locations correspond to RCA gridlines. The RCA grids provide a high density of discrete guidelines, while the puncturable device material offers a continuous workspace for potential needle insertions.

### 3.2. Phantom Study

**Figure 5:**
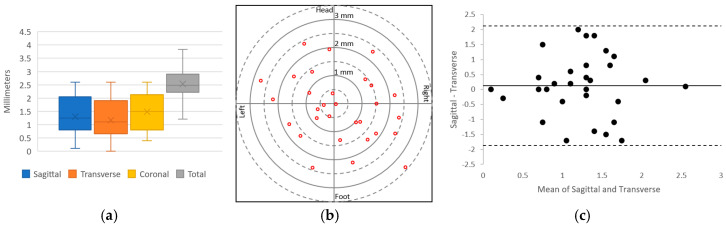
Plots of the lesion-targeting error in phantom studies. (**a**) Quartile plot of sagittal, transverse, coronal, and total targeting error. (**b**) Plot of radial error. Solid rings every millimeter, dashed rings every 0.5 mm. (**c**) Bland–Altman plot of sagittal and transverse error measurements.

The results of the needle insertion experiments performed on an abdominal phantom are displayed in Figure 5. Preliminary results showed successful CT-guided biopsy needle placements in an abdominal phantom. The mean targeting accuracy over all experiments was 2.51 mm (SD 0.59 mm, *n* = 30). Voxel size for the images used in these calculations was 0.43 mm × 0.43 mm × 1.0 mm. Figure 5a displays a box and whisker plot comparing the targeting error between the sagittal, transverse and coronal axes; Figure 5b depicts the radial error; and Figure 5c shows a Bland–Altman plot of the sagittal and transverse error measurements. The results show that the error is relatively evenly distributed around the target.

## 4. Discussion

The origami lesion-targeting device provides several potential benefits for CT-guided percutaneous biopsy. One primary advantage is that since the device is attached to the patient, it allows for the needles to move with the movement of the patient, thus potentially reducing the risk of tissue laceration by rigidly held needles. Another major benefit is that the device allows for multiple needles to be inserted, making it applicable for composite ablation using multiple electrodes. Furthermore, the device has the potential to guide off-axial needle insertion for highly inaccessible lesions that require multiple plane angulations. In addition to the potential benefits, the device can be manufactured quickly and inexpensively, making it disposable and therefore ideal for the surgical environment. 

The device does not achieve as high a degree of targeting accuracy as the state-of-the-art computer-aided and robotic navigation systems. However, it provides smaller hospitals with an effective alternative where computer-aided and robotic navigation systems are not available. Moreover, the device does not require system set-up, instrument calibration, registration, and familiarity with the system to obtain optimal accuracy, as required with computer-aided and robotic systems. 

Our preliminary evaluation exposed several limiting characteristics. The fixed nature of the needle guide may be disadvantageous in situations where the target lesion moves with time, such as with pulmonary lesions during respiration. Change to free-hand insertion as necessary may be prohibited without removing the needle from the body and detaching the device from the patient. The flat surface of the device makes it unsuitable for accessing lesions which require a lateral approach, limiting it to be used mainly for abdominal procedures. Another limitation is that the large footprint of the device can make it difficult to be used with ultrasound. In future work, we will evaluate the device with in-vivo insertion and explore options for mitigating the effects of respiratory movement of the target. We will also evaluate the ability to guide multiple needles and off-axial needle insertion. We will explore iodine-based radiocontrast agents as well as other biocompatible materials to construct the device from. Additionally, the biocompatibility and hemocompatibility regulatory issues of having the needle traverse the material also need to be certified. Given the limitations of our methodology, more rigorous testing in a specific clinical application would be necessary to compare this device with established systems.

## 5. Conclusions

A novel device for assisting with CT-guided needle insertions is presented. The device was fabricated by laser cutting the structure from a sheet of medical grade paperboard, 3D printing two radiocontrast agent grids on to the surface and folding the structure into a rectangular prism with a viewing window. The device was evaluated through CT imaging and targeting of lesions for needle insertions in an abdominal imaging phantom. The results of the lesion-targeting experiments showed a mean targeting error of 2.53 mm (SD 0.59 mm, *n* = 30). The main advantages of the device are that it attaches to the patient (potentially reducing the risk of laceration), it supports insertion of multiple needles (making it particularly suitable for composite ablations), and it can guide off-axial needle insertion. The low-cost and disposability are well-suited for interventional settings.

## Figures and Tables

**Table 1:** Variables in needle insertion location Equations (1)–(6).

Variable	Description
*T*	Target point; *T* = (*T_x_*, *T_y_*, *T_z_*)
*O*	Origin of device
*θ*	Insertion angle
*x*_1_, *x*_2_	Horizontal distance from origin to insertion locations
*z*_1_, *z*_2_	Vertical distance from top layer to bottom layer, vertical distance from bottom layer to target
*x_a_*, *x_b_*	Horizontal distance from top layer insertion location to bottom layer insertion location, horizontal distance from bottom layer insertion location to target
*d*	Needle insertion depth
